# Vaccination with Consensus H7 Elicits Broadly Reactive and Protective Antibodies against Eurasian and North American Lineage H7 Viruses

**DOI:** 10.3390/vaccines8010143

**Published:** 2020-03-23

**Authors:** Gendeal M. Fadlallah, Fuying Ma, Zherui Zhang, Mengchan Hao, Juefu Hu, Mingxin Li, Haizhou Liu, Biling Liang, Yanfeng Yao, Rui Gong, Bo Zhang, Di Liu, Jianjun Chen

**Affiliations:** 1 Department of Biotechnology, Key Laboratory of Molecular Biophysics of MOE, College of Life Science and Technology, Huazhong University of Science and Technology, Wuhan 430074, China; Gend_l78@hotmail.com (G.M.F.); mafuying@hust.edu.cn (F.M.); 2 CAS Key Laboratory of Special Pathogens and Biosafety, Chinese Academy of Sciences, Wuhan 430071, China; zherui4138@163.com (Z.Z.); hmc@wh.iov.cn (M.H.); hujuefu2018@163.com (J.H.); 2009limingxin@163.com (M.L.); liuhz@wh.iov.cn (H.L.); liangbl@wh.iov.cn (B.L.); gongr@wh.iov.cn (R.G.); zhangbo@wh.iov.cn (B.Z.); liud@wh.iov.cn (D.L.); 3 National Virus Resource Center, Chinese Academy of Sciences, Wuhan 430071, China; 4 University of Chinese Academy of Sciences, Beijing 100000, China; 5 National Biosafety Laboratory, Wuhan Institute of Virology, Chinese Academy of Sciences, Wuhan 430071, China; yaoyf@wh.iov.cn

**Keywords:** COBRA, influenza, consensus H7, broadly reactive, mice

## Abstract

H7 subtype avian influenza viruses have caused outbreaks in poultry, and even human infection, for decades in both Eurasia and North America. Although effective vaccines offer the best protection against avian influenza viruses, antigenically distinct Eurasian and North American lineage subtype H7 viruses require the development of cross-protective vaccine candidates. In this study, a methodology called computationally optimized broadly reactive antigen (COBRA) was used to develop four consensus H7 antigens (CH7-22, CH7-24, CH7-26, and CH7-28). In vitro experiments confirmed the binding of monoclonal antibodies to the head and stem domains of cell surface-expressed consensus HAs, indicating display of their antigenicity. Immunization with DNA vaccines encoding the four antigens was evaluated in a mouse model. Broadly reactive antibodies against H7 viruses from Eurasian and North American lineages were elicited and detected by binding, inhibition, and neutralizing analyses. Further infection with Eurasian H7N9 and North American H7N3 virus strains confirmed that CH7-22 and CH7-24 conferred the most effective protection against hetero-lethal challenge. Our data showed that the consensus H7 vaccines elicit a broadly reactive, protective response against Eurasian and North American lineage H7 viruses, which are suitable for development against other zoonotic influenza viruses.

## 1. Introduction

H7 subtype avian influenza virus has caused outbreaks in poultry, and even human infection, for decades. Both North American and Eurasian lineages of H7 viruses have been associated with human infection [1], and the global distribution of this subtype has affected poultry in Europe, America, Asia, and Oceania [2]. To date, human infections have been caused by the poorly pathogenic H7 subtypes, H7N2, H7N3, H7N4, H7N7, and H7N9, as well as the highly pathogenic H7N3 and H7N7 [2,3,4,5]. Before 2003, less than 20 sporadic cases of human infection with H7 viruses were reported in Europe and America [1]. In 2003, outbreaks of HPAI H7N7 occurred in poultry of several European countries, and 86 poultry workers as well as three of their family members were infected with this subtype in The Netherlands [5]. Among them, almost all infected people developed mild–moderate conjunctivitis and one person died from pneumonia and acute respiratory distress syndrome [5]. This outbreak represented the first H7 avian influenza outbreak in humans. H7N9 caused another outbreak in 2013 in China [4,6]. The viruses most likely emerged in the Yangtze River Delta region of China and disseminated widely throughout the country [7,8]. Since its emergence in 2013, the H7N9 virus has been circulating in domestic poultry in China and caused five epidemic waves of human infections, and it has evolved continually and substantially [9,10,11,12,13]. As of December 2019, 1568, laboratory-confirmed cases of human infection with H7N9 viruses, including at least 616 deaths, have been reported to the WHO [14]. 

To date, H7 influenza viruses have not caused a pandemic, but have the potential. Vaccination is an effective strategy to prevent and control enzootic influenza infection. However, antigenically distinct Eurasian and North American lineage subtype H7 viruses require the development of appropriate vaccine candidates. Currently available influenza vaccines mainly provide hemagglutinin strain-specific protection, but rarely provide cross-protection against divergent strains. It is therefore particularly important to develop an intra- and inter-subtype universal vaccine. In recent years, a methodology called computationally optimized broadly reactive antigen (COBRA) was used to design intra-subtype universal influenza vaccines [15]. COBRA employs multiple rounds of layered consensus building to generate influenza vaccine HA immunogens [16]. This strategy has been used to develop broadly protective vaccine candidates against various influenza subtypes in animal models, including enzootic highly pathogenic H5 [15], seasonal circulating H1 [17], and H3 viruses [18]. The success in preclinical data highly suggests that the COBRA strategy is flexible to develop vaccines against other subtypes of influenza viruses. 

DNA vaccines constitute a powerful alternative to conventional vaccines because they can induce both humoral and cellular immune responses. With DNA vaccinations, because the encoded protein is synthesized in its native form inside the host cell, the antibody responses induced in mice by consensus H7 DNA vaccination in our study reflected the real immunogenicity of the antigen in mice. In this study, four consensus H7 immunogens were generated based on the COBRA approach. Immunization with DNA vaccines encoding the four antigens were then evaluated in a mouse model. The mice elicited broadly reactive antibodies against H7 viruses from Eurasian and North American lineages. Further infection with Eurasian H7N9 and North American H7N3 virus strains confirmed that two consensus antigens (CH7-22 and CH7-24) conferred the most effective protection against hetero-lethal challenge.

## 2. Materials and Methods 

### 2.1. Ethics Statement

This study was reviewed and permitted by the Ethics Committee of the Wuhan Institute of Virology, Chinese Academy of Sciences. Animal experiments were carried out in an animal biosafety level 2 facility. The experiments were conducted under the Chinese national guidelines of ethics and policies for the care of laboratory animals and were certified by the Welfare and Ethical Review Board of Wuhan Institute of Virology’s Institutional Animal Care and Use Committee.

### 2.2. Cells and Viruses

Madin Darby Canine Kidney (MDCK) and BHK-21 cells were obtained from the National Virus Resource Center, Wuhan Institute of Virology, Chinese Academy of Sciences, and cultured in Dulbecco’s modified Eagle’s medium (DMEM) supplemented with 10% fetal bovine serum. The H7N9 virus used in the animal experiments was NIBRG-267 (H7N9), a vaccine strain from the National Institute for Biological Standards and Control (NIBSC) and adapted to mice [19]. H7N7 [A/Phalacrocorax carbo/Hubei/HH179/2013 (H7N7)] was isolated from wild waterfowl and described previously [20]. H7N3 [A/chicken/BC/CN006/2004 (H7N3) (rBC04/H7N3)] and H7N1 [A/Rhea/North Carolina/39482/93 (H7N1) (rNC93/H7N1)] with two surface genes of their own and six internal genes from A/Puerto Rico/8/34 were prepared in our laboratory [21]. H7N9 and H7N7 strains represent Eurasian lineage viruses, and H7N3 and H7N1 strains were North American viruses. H7N9, H7N7, H7N3, and H7N1 viruses were inoculated into 10-day-old SPF embryonated chicken eggs to propagate. The median tissue culture infective dose (TCID_50_) of the viruses was determined with eight replicates in fresh medium containing 1 μg/mL TPCK-trypsin (Sigma-Aldrich, St. Louis, USA).) [22]. 

### 2.3. Antigenic Construction and Characterization 

Human H7 HA protein sequences (*n* = 1502) were downloaded from the NCBI influenza virus resource and GISAID (Global Initiative on Sharing Avian Influenza Data) database until early 2017. A multiple alignment analysis was conducted and a maximum-likelihood phylogenic tree was generated using MEGA7 software. Consensus generation of each round was performed as described previously [18]. Briefly, for each round of consensus generation, multiple alignment was performed and the most common residue was obtained and yielded the consensus sequence. Multiple rounds of consensus assembly were layered to yield primary and secondary consensus sequences. The last outcome of amino acid sequences was applied to COBRA. Four H7 HA protein sequences were generated and named CH7-22, CH7-24, CH7-26, and CH7-28. A phylogenetic tree was inferred from hemagglutinin amino acid sequences using the maximum likelihood method, and groupings of the four H7 antigens in trees were identified using Figtree software. Furthermore, to determine whether the four sequences could form a three-dimensional conformation, homology models of CH7-22, CH7-24, CH7-26, and CH7-28 were constructed in the SWISS-MODEL webserver. The antigenic sites of H7 HA COBRA were determined based on alignment with the A/Anhui/1/2013 (H7N9) sequence as described previously [23]. Colorization of five antigenic sites (A, B, C, D and E) in trimerized H7 protein was performed using PyMol.

### 2.4. Gene Synthesis and Plasmid Construction

After designing the consensus immunogens, the four consensus sequences were subjected to codon/RNA optimization to enhance gene expression in mammal cells as described previously [24,25]. The four H7 COBRA HAs were synthesized (Sangon Biotech, Shanghai, China) and cloned into the eukaryotic expression plasmid pVAX1 (Invitrogen, San Diego, CA, USA) using XhoI and EcoRI with a Kozak translation initiation sequence (GCCACC) inserted before the start codon. The plasmids were propagated in *Escherichia coli* DH5α and purified using an E.Z.N.A.^®^ Endo-Free Plasmid Maxi Kit (Omega Bio-tek, Inc., Norcross, GA, USA). Sequencing was performed to ensure no mutations were in the plasmid. The HA genes of A/Shanghai/02/2013 (H7N9) and A/Phalacrocorax carbo/Hubei/HH179/2013 (H7N7) were amplified and cloned into pVAX1 to generate pH7N9 and pH7N7, following the same method for COBRA HAs.

### 2.5. In Vitro Expression of Consensus H7 HAs

BHK-21 cells (1 × 10^6^) were transfected with 3 μg pCH7-22, pCH7-24, pCH7-26, pCH7-28, and pVAX1 (empty vector) using Lipofectamine 3000 Transfection Reagent (Invitrogen). After incubation for 5 h, the medium was replaced with fresh medium containing 10% fetal bovine serum. At 24 h post-transfection, the cells were washed with PBS, fixed with 4% paraformaldehyde (pH 7.4) for 30 min, permeabilized with 0.2% Triton X-100 in PBS for 30 min, and then stained with Hoechst 33258 for 30 min. Indirect immunofluorescence staining was performed with monoclonal antibodies CR9114 (broad-spectrum anti-stem neutralizing antibody) [26] or P52E03 (targeting the HA head of subtype H7) [21], and then FITC-labeled goat anti-human IgG. Imaging was conducted under a fluorescence microscope (Nikon Eclipse TE2000).

### 2.6. Vaccination and Antibody Response Analysis

In vivo electroporation was carried out according to a previously described method [27,28]. Six- to 8-week-old female BALB/c mice (10 mice per group) were immunized twice with a 3-week interval. Each vaccination consisted of 30 μg pCH7-22, pCH7-24, pCH7-26, pCH7-28, pH7N9, and pH7N7 dissolved in 30 μL Tris-EDTA buffer. Control group mice were injected with PBS. After injection into the right quadricep muscle, a pair of electrode needles at 5 mm apart was inserted into the muscle to cover the DNA injection sites and electric pulses were delivered using an electric pulse generator (ECM830; BTX, San Diego, CA, USA). Two weeks after the second immunization, sera were collected from four to five mice per group and treated with a receptor-destroying enzyme (RDE) (Denka Seiken, Co., Tokyo, Japan) as described previously [21]. Briefly, the sera were treated with the RDE, followed by inactivation at 56 °C for 30 min and incubation with chicken erythrocytes to adsorb nonspecific agglutinins. The treated serum was tested in the following assays.

#### 2.6.1. Hemagglutinin Inhibition (HAI) Assay

The sera from each group of immunized or unimmunized mice were serially diluted two-fold with PBS in a 96-well polystyrene microtiter plate with 25 μL in each well. A portion of 25 μL of virus suspension containing four hemagglutinin units (HAU) of H7N9, H7N7, H7N3, and H7N1 was added to each well. After incubation of the plate at room temperature for 1 h, 50 μL of 0.5% (v/v) chicken red blood cells were added to each well, and the plate was incubated at room temperature for 30 minutes. The HAI titers were determined as the highest serum dilution that completely inhibited hemagglutination.

#### 2.6.2. Microneutralization (MN) Assay

Titers of neutralizing antibodies were determined as described previously [21]. Serum treated with the receptor-destroying enzyme (RDE) was diluted from 1:20 to 1:2560 by two-fold serial dilutions in culture medium (DMEM containing 100 U/ml penicillin G, 100 μg/mL streptomycin, and 0.5 μg/mL TPCK-treated trypsin). Diluted serum solutions were mixed with culture medium containing 100 TCID_50_ H7N9, H7N7, H7N3, and H7N1 at room temperature for 1 h. The virus-serum mix was then transferred to MDCK cells. Culture medium was added, and the plates were incubated for 72 h. Endpoints were determined by the hemagglutination titer.

#### 2.6.3. ELISA 

An ELISA was performed using a 96-well plate (EIA plate, Costar, Richmond, VA, USA) that was first coated with H7N9, H7N7, H7N3, and H7N1 (4 HAU/well) and then incubated with serial dilutions of RDE-treated serum, followed by goat anti-mouse IgG (γ-chain specific) (Southern Biotechnology Associates, Inc., Birmingham, UK) conjugated with HRP. The amount of chromogen produced was measured based on the absorbance at 420 nm using an ELISA reader (Synergy H1, Biotek, Winooski, VT, USA).

### 2.7. Adaptaion of H7N3 Influenza Virus in Mice

The mouse-adapted virus was prepared as described previously [24]. To enhance the virulence of North American lineage strain H7N3 [A/chicken/BC/CN006/2004 (H7N3) (rBC04/H7N3)], we applied 10 sequential passages in mice to achieve a mouse-adapted virus. Initially, a group of three mice was anesthetized, and each mouse was inoculated intranasally with 20 μL viral H7N3. At 3–5 days post-inoculation, mice were sacrificed and their trachea and lungs were removed. Tissues were washed twice in 2 mL phosphate-buffered saline (PBS) containing 0.1% bovine serum albumin (BSA). The bronchoalveolar washes were collected and used to infect the next batch of mice after removing cellular debris by centrifugation. Lung-to-lung passaging was repeated until the virus was lethal in mice. After 10 serial passages, mice developed clinical symptoms, including a hunched posture, weakness, weight loss, and ruffled fur, and the viruses exhibited high virulence in mice. The final adapted virus was harvested, aliquoted, and stored at −80 °C. The 50% mouse lethal dose (LD_50_) of each stock was determined using the Reed–Muench method.

### 2.8. Viral Infection and Titration

Three weeks after a boost, mice in each group (*n* = 10) were anesthetized and challenged with the mouse-adapted H7N9 and H7N3 viruses at 10 × LD_50_ by intranasal administration in 20 μL viral suspension. This infection caused rapid and widespread viral replication in the lungs and death of the unimmunized mice within 7 days. At 5 days post-infection, the trachea and lungs in each group (*n* = 4–5) were removed and washed twice by injecting 2 mL PBS containing 0.1% BSA. The bronchoalveolar wash was used for virus titration after removing cellular debris by centrifugation. The remaining mice were observed for 14 days to record survival rates and weight loss. 

Lung viral titers were determined as described previously [27]. Briefly, MDCK cells were cultured in 96-well plates overnight. After 12 h of culture, the cells were infected with 100 μL of a 1/10-dilution series of lung homogenate supernatant at 37 °C for 1 h. The supernatants were then replaced with 200 μL serum-free Dulbecco’s modified Eagle medium (DMEM) containing a cocktail of antibiotics. The cytopathic effects in the infected MDCK cells were observed daily. After incubation for 3 days, the virus titer in the supernatant was assayed by measuring hemagglutination.

### 2.9. Statistical Analysis

Viral titers were calculated by the Reed-Muench method. All data plotted with error bars are expressed as means with SD. The *p* values were generated by analyzing data with a two-tail unpaired *t* test using the Prism 8 program (GraphPad software, San Diego, CA, USA) or Mann-Whitney Test. The survival rate statistical analysis was performed with a Fisher’s exact test.

## 3. Results

### 3.1. Design of the Four Consensus H7 Proteins

**Figure 1:**
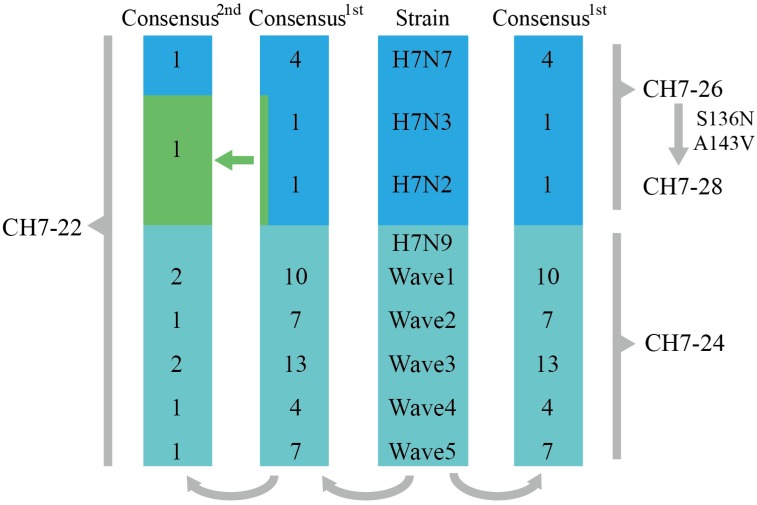
Schematic example of the layered consensus used for H7 COBRA design. The viruses were divided into four groups (H7N9, H7N7, H7N3, and H7N2) based on different HA/NA combinations. H7N9 viruses were further subdivided into five epidemic waves. Primary consensus amino acid sequences were derived from the three groups (H7N7, H7N3, and H7N2) and five subgroups (referred to five wave H7N9). CH7-26 was generated based on the primary consensus sequences of H7N7, H7N3, and H7N2. CH7-24 was obtained from the primary consensus sequences of five wave H7N9. Second consensus amino acid sequences were derived from primary sequences. Then, CH7-22 was designed based on all secondary consensus sequences. CH7-28 was derived from CH7-26 sequences by introducing S136N and A143V mutations. The green arrow refers to the secondary consensus, which was generated by alignment of the primary consensus of H7N3 and H7N2.

We applied the COBRA strategy to develop cross-reactive antigens against H7 subtype influenza viruses. Eurasian and North American lineage human H7 viruses were downloaded and aligned. The viruses were divided into four groups (H7N9, H7N7, H7N3, and H7N2) based on the different HA/NA combinations. The H7N9 viruses were further subdivided into five epidemic waves (Figure 1). Primary consensus amino acid sequences were derived from the three groups (H7N7, H7N3, and H7N2) and five subgroups (referred to five wave H7N9) (Figure 1). CH7-26 was generated based on the primary consensus sequences of H7N7, H7N3, and H7N2. CH7-24 was obtained from the primary consensus sequences of five wave H7N9. Second consensus amino acid sequences were derived from primary sequences (Figure 1). Then, CH7-22 was designed based on all secondary consensus sequences. CH7-28 was derived from CH7-26 sequences by introducing S136N and A143V (with signal sequences) mutations based on the analysis showing that both mutations emerged in H7N9 HA since the second wave (data not shown).

### 3.2. Characterization of the Four Consensus H7 Proteins

**Figure 2:**
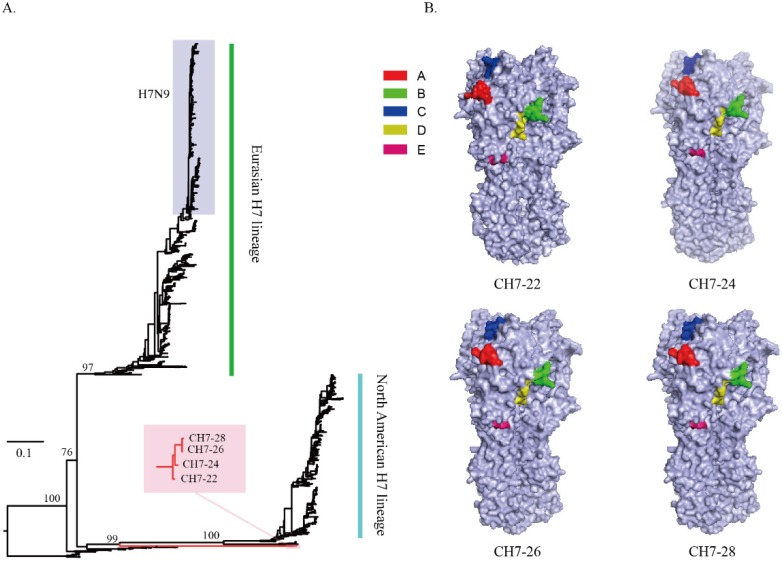
Phylogenetic analysis of the consensus H7 (**A**). A phylogenetic tree was inferred from the hemagglutinin amino acid sequences of H7 viruses (*n* = 1502) using the maximum likelihood method, and groupings of the four consensus H7 antigens in trees were identified using Figtree software. The four consensus H7 sequences with all H7 HA proteins showed that the four consensus H7 sequences (red branch) were located on separate branches without grouping with any viruses of Eurasian and North American H7 lineages. Schematic of the three-dimensional structure of four trimerized consensus H7 proteins (**B**). After four consensus H7 sequences were constructed, homology models were created for each one using the SWISS-MODEL webserver. The antigenic sites of consensus H7 HA were identified by alignment with the A/Anhui/1/2013 (H7N9) sequence. The A site is shown in red, the B site in green, the C site in blue, the D site in yellow, and the E site in medium violet-red.

**Figure 3:**
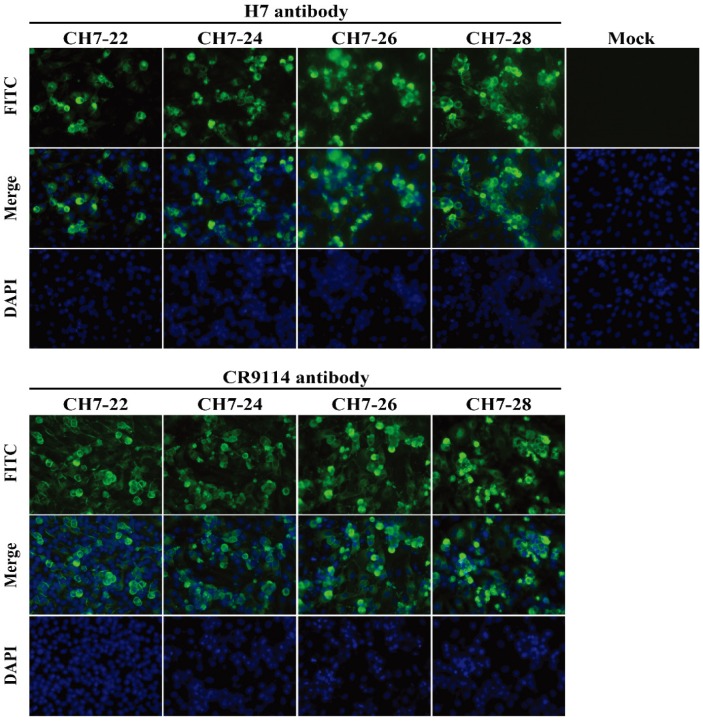
In vitro expression of consensus H7 HAs. BHK-21 cells (1 × 10^6^) were transfected with 3 μg pCH7-22, pCH7-24, pCH7-26, pCH7-28, and pVAX1 (empty vector) using Lipofectamine 3000 Transfection Reagent (Invitrogen). After incubation for 5 h, the medium was replaced with fresh medium containing 10% fetal bovine serum. At 24 h post-transfection, the cells were washed with PBS, fixed with 4% paraformaldehyde (pH 7.4) for 30 min, permeabilized with 0.2% Triton X-100 in PBS for 30 min, and then stained with Hoechst 33258 for 30 min. Indirect immunofluorescence staining was performed with monoclonal antibodies CR9114 (broad-spectrum anti-stem neutralizing antibody) or P52E03 (targeting the HA head of subtype H7) and then FITC-labeled goat anti-human IgG. Imaging was conducted under a fluorescence microscope (Nikon Eclipse TE2000).

Sequence alignment of the four consensus antigens showed that the sequence identities ranged from 97% to 98.8%. BLAST analysis of CH7-22, CH7-24, CH7-26, and CH7-28 sequences indicated that each sequence was unique, and the identities between any one of the four sequences and sequences deposited in NCBI range from 97% to 98% (data not shown). Phylogenetic analysis of the four consensus H7 sequences with all H7 HA proteins showed that the four COBRA were located on separate branches without grouping with any viruses of Eurasian and North American H7 lineages (Figure 2A). Using predictive structural modelling of COBRA H7 HA sequences, three-dimensional trimerized HA proteins were visualized (Figure 2B). In addition, a similar location of putative antigenic sites (A–E) of H7 HA was observed in the four COBRA sequences (Figure 2B), suggesting the consensus H7 HA maintained the native three-dimensional conformation and antigenic sites. Expression of the four COBRA H7 HA proteins was confirmed by an indirect immunofluorescence assay. BHK-21 cells transiently transfected with pCH-22, pCH-24, pCH-26, and pCH-28 were stained with stem-specific CR9114 or head-specific P52E03 mAbs. Binding of CR9114 or P52E03 to the four proteins was detected (Figure 3). Overall, we confirmed that the four COBRA H7 HA proteins were bound by either stem-specific or head-specific mAbs, indicating preservation of antigenic sites.

### 3.3. DNA Vaccines Encoding Consensus H7 Proteins Elicit Broadly Reactive Antibody Responses in Mice

**Figure 4:**
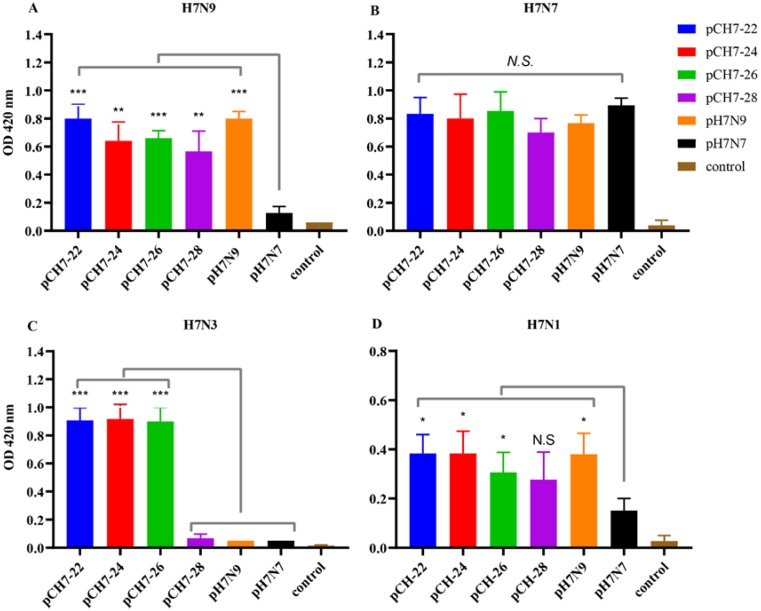
DNA vaccines encoding consensus H7 proteins elicit broadly reactive antibody responses in mice. Six to 8-week-old female BALB/c mice (10 mice per group) were immunized twice with a 3-week interval. Each vaccination consisted of 30 μg pCH7-22, pCH7-24, pCH7-26, pCH7-28, pH7N9, and pH7N7 dissolved in 30 μL Tris-EDTA buffer. Two weeks after the second immunization, sera were collected from four to five mice per group and treated with an RDE. The treated serum was tested for the titer of total IgG antibodies. An ELISA was performed using a 96-well plate coated with H7N9 (**A**), H7N7 (**B**), H7N3 (**C**), and H7N1 (**D**) (4 HAU/well), followed by incubation with serial dilutions of RDE-treated serum and then goat anti-mouse IgG (γ-chain specific) conjugated with HRP. The amount of chromogen produced was measured at 1: 100 serum dilution based on the absorbance at 420 nm using an ELISA reader (Synergy H1, Biotek). The data are shown as the mean antibody titers of four mice in each group with standard errors (error bars). Statistical significance was analyzed by *t* test. *p* values shown in bar charts and N.S. indicates no significance between two compared groups. * *p* < 0.05, ** *p* < 0.01 and *** *p* < 0.001 between indicated groups.

Upon verifying expression of all four consensus H7 constructs, we first examined the capacity of these immunogens to induce immune responses in mice against antigenic and genetically diverse Eurasian and North American lineages H7 viruses. Adult female BALB/c mice were immunized twice with DNA vaccines encoding CH7-22, CH7-24, CH7-26, and CH7-28, their serum was collected and analyzed by ELISA at 2 weeks after the second immunization. As a comparison, plasmids encoding HA proteins of H7N9 and H7N7 were immunized in parallel. The serum was tested against Eurasian H7N9 and H7N7, and North American H7N3 and H7N1 viruses. The results showed that CH-22, CH-24, and CH-26 elicited high antibody titers against all tested H7 viruses, whereas CH-28 elicited relatively low titers against H7N3 (Figure 4). These results indicated that CH7-22, CH7-24, and CH7-26 induced cross-reactive anti-H7 antibodies.

### 3.4. DNA Vaccines Encoding COBRA H7 Proteins Elicit Broadly Neutralizing Antibody Responses in Mice

**Figure 5:**
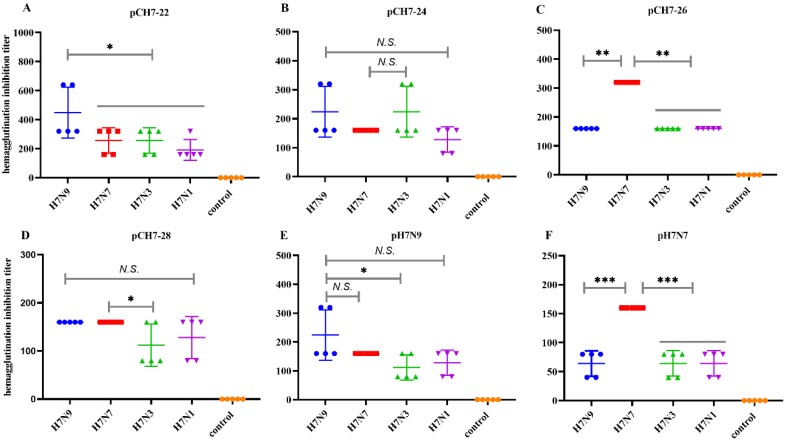
DNA vaccines encoding consensus H7 proteins elicit broad HAI antibody responses in mice. Sera from each group of immunized and unimmunized mice were serially diluted by two-fold with PBS in a 96-well polystyrene microtiter plate with 25 μL in each well. A portion of 25 μL virus suspension containing 4 hemagglutinin units (HAU) H7N9, H7N7, H7N3, and H7N1) were added to each well. After incubation of the plate at room temperature for 1 h, 50 μL of 0.5% (v/v) chicken red blood cells were added to each well, and the plate was incubated at room temperature for 30 minutes. HAI titers were determined as the highest serum dilution that completely inhibited hemagglutination. Values are the mean titers plus the standard error of the mean (error bars) (**A**–**F**). Statistical significance was analyzed by *t* test or Mann-Whitney Test. *p* values shown in bar charts and N.S. indicates no significance between two compared groups. * *p* < 0.05, ** *p* < 0.01 and *** *p* < 0.001 between indicated groups.

**Figure 6:**
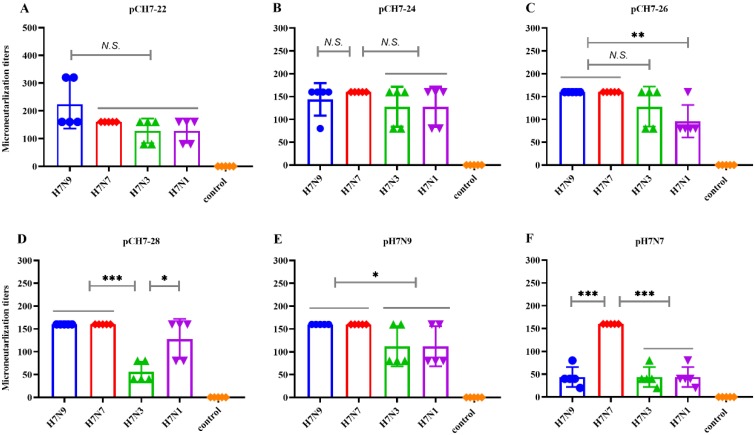
DNA vaccines encoding consensus H7 proteins elicit broad neutralizing antibody responses in mice. Serum treated with a receptor-destroying enzyme (RDE) was diluted from 1:20 to 1:2560 by two-fold serial dilutions in culture medium (DMEM containing 100 U/mL penicillin G, 100 μg/mL streptomycin, and 0.5 μg/mL TPCK-treated trypsin). Diluted serum solutions were mixed with culture medium containing 100 TCID_50_ H7N9, H7N7, H7N3, and H7N1 at room temperature for 1 h. The virus-serum mix was then applied to MDCK cells. Culture medium was added, and the plates were incubated for 72 h. Endpoints were determined by the hemagglutination titer. Values are the mean titers plus the standard error of the mean (error bars) (**A**–**F**). Statistical significance was analyzed by *t* test. *p* values shown in bar charts and N.S. indicates no significance between two compared groups. * *p* < 0.05, ** *p* < 0.01 and *** *p* < 0.001 between indicated groups.

We further investigated whether the elicited antibodies had the potential to cross-neutralize H7 viruses. As shown in Figure 5, each consensus immunogen provoked effective high HAI titers against four tested viruses. Mice that received pCH7-22 had HAI titers against H7N9 of ≥1:320 and ≥1:160 titers against H7N7, H7N3, and H7N1 viruses. The mice that received pCH7-24 had titers of ≥1:160 against H7N9, H7N7, and H7N3 viruses, and ≥1:80 titers against H7N1. The HAI titer in mice vaccinated with pCH7-26 was 1:160 against H7N9, H7N3, and H7N1 viruses, and 1:320 against H7N7. Mice vaccinated with pCH7-28 had a slightly lower HAI titer against H7N3 and H7N1 (titers ≥1:80) compared with H7N9 and H7N7 viruses (≥1:160). The mice vaccinated with pH7N9 and pH7N7 had apparently lower cross-reactive HAI titers against heterologous strains than those against homologous viruses. Overall, these data confirmed that the DNA vaccine encoding CH7-22, CH7-24, and CH7-26 elicited a better broad cross-reactive HAI against divergent H7 viruses. Next, we examined the cross-neutralizing activity against H7N9, H7N7, H7N3, and H7N1 viruses. The results further confirmed that plasmids encoding CH7-22, CH7-24, and CH7-26 elicited a better broad cross-reactive HAI against divergent H7 viruses (Figure 6).

### 3.5. DNA Vaccination with Consensuses H7 Confers Protection against Lethal H7N9 Influenza Virus Challenge in Mice

**Figure 7:**
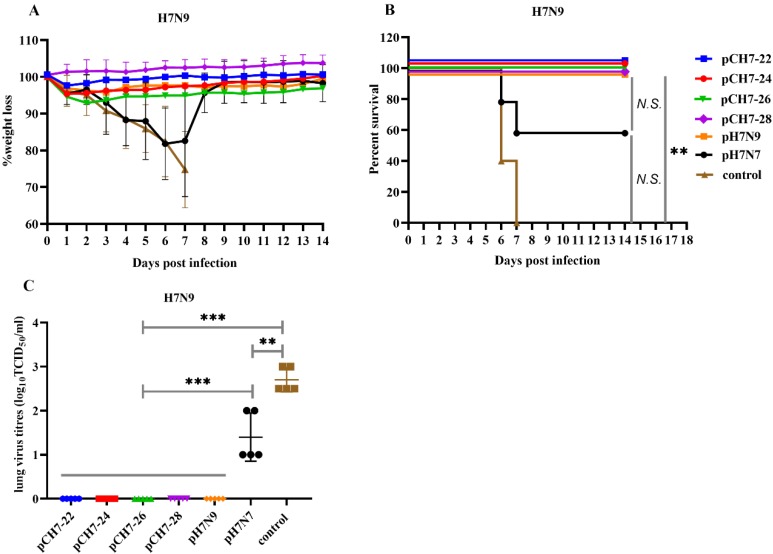
DNA vaccination with consensuses H7 confers protection against lethal H7N9 influenza virus challenge in mice. Groups of mice were vaccinated twice with pCH7-22, pCH7-24, pCH7-26, pCH7-28, pH7N7, or pH7N9 and challenged with 10 × LD50 A/Shanghai/2/2013/H7N9 virus at 3 weeks after the second immunization. Changes in body weight (**A**) and survival rates (**B**) were monitored daily for 2 weeks post-infection. Four mice from each group were sacrificed at day 5 post-infection, and lungs were collected for virus titration (**C**). The statistical analysis of survival rate difference between groups was performed with a Fisher’s Exact test. The statistical analysis of viral titers was analyzed by *t* test. *p* values shown in bar charts and N.S. indicates no significance between two compared groups. ** *p* < 0.01 and *** *p* < 0.001 between indicated groups.

After evaluating the cross-reactive antibody responses elicited by the consensus H7 against Eurasian and North American lineage H7 viruses, we investigated the efficacy of the consensus H7 in a mouse model. Groups of mice were vaccinated twice with pCH7-22, pCH7-24, pCH7-26, pCH7-28, pH7N7, or pH7N9 and then challenged with 10 × LD_50_ A/Shanghai/2/2013/H7N9 virus at 3 weeks after the second immunization. Four mice from each group were sacrificed at day 5 post-infection and their lungs were collected for virus titration. Changes in body weight and survival rates were monitored daily for 2 weeks post-infection. Mice that lost more than 25% of their body weight were euthanized according to guidelines. Mice vaccinated with pCH7-22, pCH7-24, pCH7-26, pCH7-28, or pH7N9 only showed slight weight loss at 3 dpi, recovered quickly, and all survived within 2 weeks (Figure 7). Mice vaccinated with pH7N7 survived at a rate of 60% and their weight decreased significantly, reaching maximum loss at 7 dpi. Mice in the control group died within 7 days of the infection. Consistent with the survival rate and weight loss, high lung viral titers were detected in pH7N7 and control groups, whereas no virus was detected in mice vaccinated with pCH7-22, pCH7-24, pCH7-26, pCH7-28, or pH7N9 (Figure 7). These results suggest that the consensus H7 provides protection against lethal H7N9 infection in a mouse model.

### 3.6. DNA Vaccines Elicit Protection against H7N3 Influenza Virus Infection

**Figure 8:**
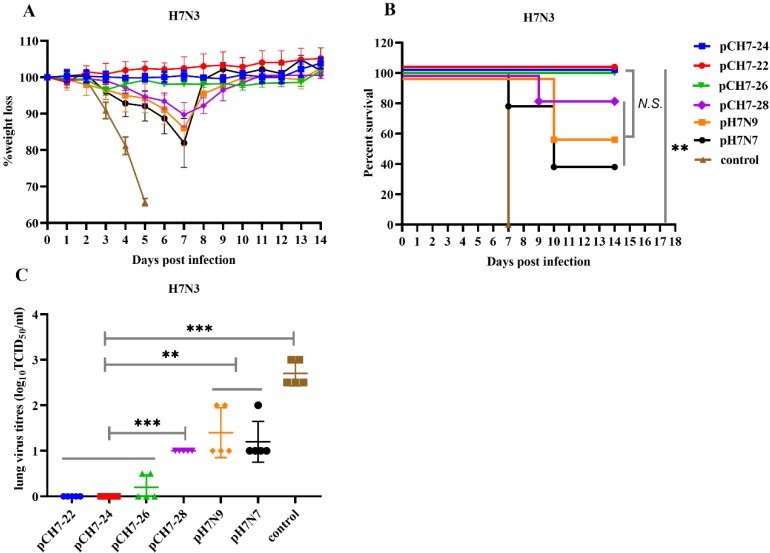
DNA vaccination with consensus H7 confers protection against lethal H7N3 influenza virus challenge in mice. Groups of mice were vaccinated twice with pCH7-22, pCH7-24, pCH7-26, pCH7-28, pH7N7, or pH7N9 and challenged with 10 × LD50 mouse-adapted A/Chicken/BC/CN006/2004 (H7N3) from the North American lineage virus at 3 weeks after the second immunization. Changes in body weight (**A**) and survival rates (**B**) were monitored daily for 2 weeks post-infection. Four mice from each group were sacrificed at day 5 post-infection, and lungs were collected for virus titration (**C**). The statistical analysis of survival rate difference between groups was performed with a Fisher’s Exact test. The statistical analysis of viral titers was analyzed by *t* test. *p* values shown in bar charts and N.S. indicates no significance between two compared groups. ** *p* < 0.01 and *** *p* < 0.001 between indicated groups.

Next, avian influenza strain A/Chicken/BC/CN006/2004 (H7N3) from the North American lineage was used to test the cross-protective efficacy of pCH7-22, pCH7-24, pCH7-26, pCH7-28 pH7N7, or pH7N9. Mice were vaccinated as described above. At week 6, the mice were infected with a lethal dose of mouse-adapted A/Chicken/BC/CN006/2004/H7N3 (10 × LD_50_). Mice vaccinated with pCH7-22, pCH7-24, and pCH7-26 all survived and showed no symptoms of morbidity or weight loss within 2 weeks (Figure 8). However, the mice that received pCH7-28, pH7N7, and pH7N9 had apparent weight loss and only showed partial protection against lethal H7N3 infection with survival rates of 83%, 40%, and 60%, respectively (Figure 8). After 3 days of infection, body weight in the control group decreased rapidly, and all mice died at 5 dpi. Consistent with the survival rate and weight loss, no lung viruses were detected in mice vaccinated with pCH7-22 or pCH7-24 at 5 dpi. Animals vaccinated with either pCH7-26, pCH7-28-H7, pH7N7, or pH7N9 had detectable viral titers (Figure 8). However, the control group had high levels of lung viral titers compared with vaccinated groups.

## 4. Discussion

Vaccination is the most effective countermeasure to prevent the threat of influenza viruses. Among viral proteins of the influenza virus, HA is the main protein used as a vaccine candidate because it elicits a highly protective immune response in the host [29,30]. However, under selective pressure from the host immune system, evolution of HA protein antigenicity has resulted in escape of antibody recognition [29]. Consequently, yearly updated influenza vaccines consist of inactivated virus with HA from types A(H1N1) pdm09 and A(H3N2), and one/two type B that best match the predicted circulating strains, but lack cross-protection against novel variants [31]. It is currently impossible to predict which antigenic variants may emerge, and novel vaccine candidates are needed to elicit broad spectrum immune responses against variants. Recently, a new technology called COBRA was used to design consensus antigens of various subtypes including H1, H3, and H5. COBRA consensuses have been generated using HA protein sequences of H5N1 and vaccination elicits broad antibody responses [15]. Nine prototypes of H1N1 were evaluated in mice as virus-like particles that provoked cross-reactive immune responses against the divers of H1N1 viruses [17]. In addition, a previous study confirmed that the COBRA method is an effective strategy to develop an H3N2 universal vaccine [18]. Collectively, these findings highly suggest that COBRA is a powerful method to generate subtype universal vaccines. 

In this study, four consensus H7 antigens (CH7-22, CH7-24, CH7-26, and CH7-28) were designed by COBRA and generated. In vitro experiments confirmed that head-specific P52E03 and stem-specific CR9114 mAbs bound the cell surface-expressed consensus HAs, indicating retention of the antigenicity of the four consensus HAs. It has been shown that COBRA-designed consensus proteins retain highly immunogenic and cross-reactive epitopes [32]. Consistent with the in vitro experiments, when applied as DNA vaccines in BALB/c mice, broadly reactive antibodies against H7 viruses from Eurasian and North American lineages were elicited and detected by inhibition, binding, and neutralizing analyses. An ideal universal influenza vaccine needs to elicit broad spectrum reactive antibodies against intra- and inter-subtype viruses. Previous studies have described that consensus HA proteins induce broadly subtype-specific neutralizing antibodies against H1, H3, and H5 HAs [15,17,18]. These results combined with our data highly suggest that using COBRA to design consensus HAs is an applicable method to develop universal vaccines for not only these subtypes, but also H7 and even other subtypes. 

In viral infection experiments, protection against lethal H7N9 viral challenge was also provided by DNA immunization with pCH7-22, pCH7-24, pCH7-26, or pCH7-28. The viral titers were undetectable in the lungs of consensus DNA-immunized mice at 5 dpi, suggesting that the consensus H7 vaccines that we designed and tested effectively prevented lethal H7N9 infection in mice. The clinical outcomes of mice immunized with consensus H7 were consistent with the antibody response. The four consensus H7 DNAs induced higher IgG antibody, HAI, and neutralizing antibody titers against H7N9 than pH7N7 and induced a similar high titer compared with pH7N9. Therefore, a lower survival rate, more weight loss, and higher lung viral titers were observed in the pH7N7-immunized group. The H7N9 virus infected humans in 2013 [4], resulting in 1567 cases with at least 615 deaths as of 5 September 2018 [33]. The 2016–2017 epidemic was the fifth wave and caused the largest human infection with 758 cases and 288 deaths [34,35]. Evolutionary analysis of H7N9 viruses revealed two outbreak sources of H7N9, the Yangtze and Pearl River Delta regions [36]. Importantly, highly pathogenic H7N9 viruses emerged in the fifth wave [37]. The continuous evolution of H7N9 subtype viruses poses a long-term threat to public health and requires a universal vaccine. In the future, the consensus H7 proteins, especially CH7-22, CH7-24, and CH7-26, have the potential for development of a vaccine candidate against diversified H7N9 viruses.

We next tested the efficacy of the consensus H7 proteins against American lineage H7N3. After challenge with a lethal H7N3 virus, the results showed that all mice vaccinated with pCH7-22, pCH7-24, and pCH7-26 survived and showed no symptoms of morbidity or weight loss within 2 weeks. Consistent with the survival rate and weight loss, no lung viruses were detected in mice vaccinated with pCH7-22 or pCH7-24 at 5 dpi. Mice that received pCH7-28, pH7N7, and pH7N9 had apparent weight loss and only showed partial protection against lethal H7N3 infection with survival rates of 83%, 40%, and 60%, respectively. CH7-28 was derived from CH7-26 sequences by introducing S136N and A143V mutations based on the analysis showing that both mutations emerged in H7N9 HA since the second wave. The introduction of the two mutations may affect the conformational epitopes and subsequently result in less cross-neutralizing, since the antibody titers against American lineage H7N3 (ELISA, HAI, or MN analysis) in CH-28 immunized group were apparently lower than in the other three consensus H7 protein immunized groups. Based on these results, consensus CH7-22 and CH7-24 proteins showed better efficacy against American lineage H7N3 than the other consensus proteins. Geographical separation of the host species has shaped H7 AIVs into independently evolving Eurasian and American lineages [38]. Among each lineage, H7 AIVs circulating in migratory birds have conserved antigenic epitopes and efficient cross-reactivity with each other. Although previous studies have shown that vaccinations with divergent heterologous H7 immunogens from both Eurasian and American lineages raise cross-reactive HAI antibodies against H7N9 viruses and even protect mice from lethal viral infections [39,40,41,42], few studies have described the result of immunization with a Eurasian lineage H7 vaccine against an American lineage virus. Here, we found that Eurasian lineage pH7N9 and pH7N7 vaccination of mice had less efficacy against American lineage H7N3 viral infection compared with consensus pCH7-22 or pCH7-24 proteins. These results highly suggested that both CH7-22 and CH-24 consensus antigens are ideal universal vaccines against divergent H7 viruses. In the future, we need to evaluate the efficacies of pCH7-22 and pCH7-24 against other Eurasian and American lineage H7 viruses. Furthermore, a large variety of AIVs (H5, H7, H9 and other subtypes) circulate in poultry in countries worldwide. This study will help develop universal vaccine against AIVs and contribute to prevent and control AIVs in poultry.

## 5. Conclusions

In this study, we describe development of a highly effective consensus H7 HA using the COBRA method, which stimulates robust cross-reactivate immune responses. Two (CH7-22 CH7-24) out of four consensus H7 antigens have the potential for development as a H7 universal vaccine candidate. These results also highly suggest that using COBRA to design consensus HAs is an applicable method to develop universal vaccines for not only H7 subtypes, but also other subtypes (for human, poultry, or both).
